#  Associations of Prenatal Growth with Metabolic Syndrome, Insulin Resistance, and Nutritional Status in Chilean Children

**DOI:** 10.1155/2014/472017

**Published:** 2014-06-15

**Authors:** Francisco Mardones, Pilar Arnaiz, Paz Pacheco, Angelica Dominguez, Luis Villarroel, Johan G. Eriksson, Salesa Barja, Marcelo Farías, Oscar Castillo

**Affiliations:** ^1^Department of Public Health, Faculty of Medicine, Pontificia Universidad Católica de Chile, Santiago, Chile; ^2^Department of Pediatrics, Faculty of Medicine, Pontificia Universidad Católica de Chile, Santiago, Chile; ^3^Department of Nutrition, Diabetes and Metabolism, Magister of Nutrition Program, Faculty of Medicine, Pontificia Universidad Católica de Chile, Santiago, Chile; ^4^Unit of General Practice, Helsinki University Central Hospital, Helsinki, Finland; ^5^Department of General Practice and Primary Health Care, University of Helsinki, Helsinki, Finland; ^6^Folkhalsan Research Centre, Helsinki, Finland; ^7^Department of Obstetrics and Gynecology, Faculty of Medicine, Pontificia Universidad Católica de Chile, Santiago, Chile; ^8^School of Nutrition, Faculty of Medicine, Pontificia Universidad Católica de Chile, Santiago, Chile

## Abstract

*Introduction*. The association of prenatal growth with nutritional status, metabolic syndrome (MS), and insulin resistance (IR) was studied in school-age children. *Methods*. A retrospective cohort study was designed linking present data of children with perinatal records. 3325 subjects were enrolled. Anthropometry, blood pressure (BP), and pubertal status were assessed. Blood lipids, glucose, and insulin were measured. Linear associations were assessed using the Cochran-Armitage test. Odds ratios and nonlinear associations were computed. *Results*. 3290 children (52% females, mean age of 11.4 ± 1 years) were analyzed. Prevalence of obesity, stunting, MS, and IR was 16.0%, 3.6%, 7.3%, and 25.5%, respectively. The strongest positive association was between birth weight (BW) and obesity (OR 2.97 (95% CI 2.01–4.40) at BW ≥ 4,000 g compared to BW 2,500–2,999). The strongest inverse association was between birth length (BL) and stunting (OR 8.70 (95% CI 3.66–20.67) at BL < 48 cm compared to BL 52-53 cm). A U-shaped association between BL and BP ≥ 90th percentile was observed. Significant ORs were also found for MS and IR. Adjustments for present fat mass increased or maintained the most prenatal growth influences. *Conclusions*. Prenatal growth influences MS, IR, and nutritional status. Prenatal growth was more important than present body composition in determining these outcomes.

## 1. Introduction

Nutritional conditions during pregnancy, infancy, and childhood have been proposed to have a major impact on the development of obesity and related metabolic traits such as metabolic syndrome (MS) and insulin resistance (IR) [1–4].

Birth weight (BW), placental weight, and measures of size at birth have all been related to risk factors for chronic disease in adult life [2, 5]. Birth length (BL) has consistently been associated with adolescent and adult height [6–8]. Shorter than average adults are at a higher risk for obesity and are also more susceptible to diabetes and CVD, independent of BMI; in contrast, taller children have a higher risk for obesity [2, 9]. Obesity in childhood has consistently been related in young adults to blood pressure (BP), insulin resistance (IR), and lower high-density lipoprotein-cholesterol (HDL-C) concentration; cardiovascular risk profile may be especially unfavorable in children with increased waist circumference (WC) [2]. A recent report based upon the same sample of children as the present study has confirmed strong associations between obesity and BP, IR, HDL, WC, triglycerides (TG), and the MS [10]. Gestational age (GA) is an important factor and premature births have been associated with increased BP and IR [11]; the incidence of prematurity is increasing worldwide [12].

The MS is a set of risk factors that may appear during childhood and have been closely linked to IR [13]. Obesity, especially of the abdominal type, and a sedentary lifestyle are important contributors to the development of IR.

We recently studied 2,152 Chilean schoolchildren aged 10–15 years, assessing possible associations between MS and IR to prenatal growth; however, nutritional status indicators were not included in those analyses [14]. The main results of that study showed that nonoptimal prenatal growth seemed to predispose to IR, high WC, and elevated BP in children.

This study aimed to investigate the associations of prenatal growth with MS and IR, including indicators of nutritional status, using a larger sample size than the previously reported.

## 2. Material and Methods

### 2.1. Design

A retrospective cohort study of school-aged children, from 20 public schools managed by the Municipality of Puente Alto, Santiago, Chile, was designed. The public schools reach nearly 40% of all primary schools in Chile [15]. All children attending 5th and 6th grade of primary education were asked to participate in the study during the years 2009–2011. The national individual identification number was used to link information gathered at school with perinatal data collected by the civil registry and subsequently published by the National Institute of Statistics [16].

### 2.2. Perinatal Variables

In addition to sex, perinatal data used in this study were birth weight (BW), birth length (BL), and GA. They were determined at the maternity hospitals immediately after delivery by trained personnel [17, 18]. Infants were weighed using a 1583 Tanita electronic scale (Tanita Corporation, Arlington Heights, IL) with an accuracy of 10 g or an electronic scale, Seca 345, the accuracy being 20 g (Secacorp, Hamburg, Germany). Crown-heel length was measured using an infantometer, to the nearest 1 mm. GA was estimated as completed weeks of gestation using the date of the last menstrual period; when this data was inaccurate an early obstetric ultrasound was used. This ultrasound examination is available for most women before 20 weeks of pregnancy in Chile and most of these scans are performed with a Voluson 730 ultrasound system (GE Healthcare, Chalfont St. Giles, UK) or a Acuson 120XP (Acuson Inc., Mountain View, CA) [17]. When ultrasound examinations were not available before 20 weeks of pregnancy, GA was usually estimated by a postnatal clinical examination of the newborns performed by the physician in charge. Preterm delivery was defined as GA ≤ 37 weeks [19].

### 2.3. Metabolic Syndrome

The criteria of Cook et al. were used to define MS in the children, when at least three out of five of its components were present, as defined by the following cut-off points [20]: WC ≥ 90th percentile [21], BP, either systolic (SBP) or diastolic (DBP) ≥ 90th percentile [22], low HDL-C ≤ 40 mg/dL, TG ≥ 110 mg/dL, and glucose (GLU) ≥ 100 mg/dL. Recently proposed local cut-off points of homeostasis model assessment index (HOMA), according to sex and pubertal maturation, were used to classify IR [23].

### 2.4. Anthropometry

The evaluations at each school were performed by a trained nurse and a nutritionist. Height and weight were measured using a stadiometer and a beam-scale Seca, with an accuracy of 50 g, while being barefoot and lightly clothed. The final height and weight were the respective averages of three measurements. We calculated body mass index (BMI = weight in kg/height^2^ in m) expressed in percentiles and* z*-scores [24]. Nutritional status was classified according to BMI percentiles as normal: 5 to 84, overweight: 85 to 94, obese: ≥95, and underweight: <5; short stature, or stunting, was defined as stature-for-age <5th percentile [24]. WC was measured with an inextensible tape on the upper lateral border of the right ilium in the midaxillary line at the end of an exhalation [25]; two measurements were averaged and we used the ≥90th percentile as cut-off value [21]. The triceps and subscapular skin folds were measured with a Harpenden caliper using a standard technique [25]; both were used to calculate the percentage of fat mass (%FM) using Slaughter equations [26], previously validated in Chilean children [27, 28]. A Critikon Dinamap Pro 100 blood pressure monitor was used according to international norms and the averages of three measurements of SBP and DBP were obtained and classified as abnormal using the ≥90 percentile of the same reference [22]. A voluntary private self-report of pubertal status was requested by observation of standardized photos of breast development in girls and genitalia in boys, including the presence of pubic hair [29].

### 2.5. Blood Samples

Subjects were instructed to fast (water was allowed) for 12 hours prior to drawing of blood; noncompliers were asked to return another day for the blood sampling. A single laboratory at our university was used for all blood analyses. This laboratory has been credited by the standard ISO 15189 for medical laboratories [30].

Venous blood samples were collected for determination of glucose (Gluco-quant method, glucose/hexokinase, Roche Diagnostics GmbH, Mannheim, Germany) and insulin (immunoassay direct luminometer chemotherapy, ADVIA Centaur XP. Bayer HealthCare LLC, Kyowa Medex Co., Japan); this method measures concentrations of insulin from 0.5 to 300 mUI/mL (sensitivity of 0.5 mUI/mL) with a coefficient of variation of 3.48% and 6.17% for concentrations of 23.51 mUI/mL and 62.49 mUI/mL, respectively. The formula developed by Matthews et al. [31] was used to calculate HOMA = (glucose [mmol/liter] × insulin [mUI/mL]/22.5) and to estimate IR [23]. TG and HDL-C were analyzed on the Modular Analytics P-800 platform (Roche Diagnostics GmbH, Mannheim, Germany).

### 2.6. Socioeconomic Status

Socioeconomic status was estimated using as a proxy the number of maternal years of formal education as collected at birth by the civil registry [16]. This indicator has been considered closely related to the actual socioeconomic status and the access to health services in many studies [15, 32, 33]. Maternal level of education was classified in the following way: (a) basic: 0–8 years of education; (b) medium: 9–12 years of education; and (c) upper: 13 and more years of education.

### 2.7. Statistical Analysis

The prevalence of MS and its components were described as number of cases and percentage, while perinatal variables used mean and standard deviation (SD). To assess differences by sex, Pearson's chi-square test for proportions and independent samples Student's* t*-test for averages were used. Prevalence of MS and its components was computed at each category of the perinatal variables. The presences of linear trends in this prevalence were assessed using the Cochran-Armitage test, while nonlinear associations were evaluated allowing quadratic terms in a logistic regression. Multivariate analysis was performed using logistic regression allowing specific adjustments for sex, Tanner stage, and %FM; those factors could be confounders which potentially influence the dependent variables [13, 15, 23, 34]. Crude and adjusted odds ratios (ORs) were computed, with their 95% confidence intervals (95% CI).

All data analyses were performed using 2-sided* P* values and values ≤ 0.05 were considered statistically significant. SAS software version 9.1 was used to compute statistical analysis.

### 2.8. Ethics

Parents or their representatives signed an informed consent form and boys/girls an informed acceptance form. The study was approved by the Ethics Committees of the School of Medicine (Pontificia Universidad Católica de Chile) and the National Fund for Science and Technology (FONDECYT).

## 3. Results

Initially 5,614 subjects were eligible for the study, 2,616 females and 2,998 males. A total of 3,325 children and adolescents who presented the signed informed consent forms were evaluated and 2289 refused to participate; from the latter 34.5% were females and 47.4% males (*P* < 0.001). Complete information on studied perinatal variables was obtained for 3290 children (98.9%), of whom 52% were females. There were no significant differences between those who participated or those who did not in terms of age, years of maternal education, BW, and BL.

Based on maternal education attainment, the socioeconomic status distribution in the total group of eligible subjects was medium 60.1%, basic 33.0%, and superior 6.9%. Proportions in the actually studied subjects were similar: 60.9%, 32.5%, and 6.6%, respectively. There were no major associations observed with the study outcomes except for short stature which was slightly more frequent for basic education than the other categories of maternal education (*P* = 0.039) (data not presented).

In the studied sample, mean age was 11.4 ± 1 years (range 10–15 years) being higher in males than in females. Most anthropometric characteristics assessed were slightly higher among females; only BMI (*z*-score) did not differ by sex. Among boys, mean BW and BL were higher than in females (Table 1).

Prevalence of obesity, stunting, MS, and IR was 16.0%, 3.6%, 7.3%, and 25.5%, respectively (Table 2). Obesity was more frequent in males but stunting was more frequent in females. The prevalence of MS was higher in females; IR did not differ between the genders. In the study cohort TG ≥ 110 mg/dL was the most common component of the MS, followed by WC ≥ 90th percentile. Interestingly, 99% of cases having BP ≥ 90th percentile came from SBP ≥ 90th percentile.

**Figure 1:**
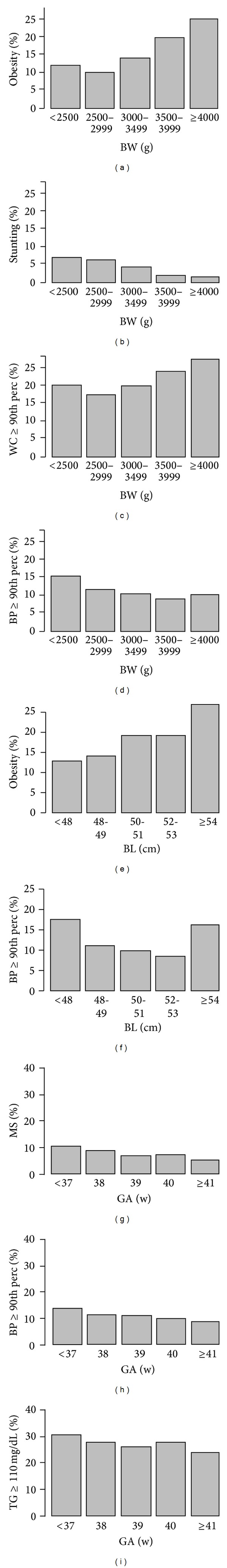
Association of perinatal variables and outcomes at 10–15 years of age, Puente Alto, Chile, 2009–2011. Association of BW with obesity ((a)* P* for trend <0.0001), stunting ((b)* P* for trend <0.0001), WC ≥ 90th percentile ((c)* P* for trend =0.0069), and BP ≥ 90th percentile ((d)* P* for trend =0.0176). Associations of BL with obesity ((e)* P* for trend =0.0027) and BP ≥ 90th percentile ((f) *P* = 0.0002 linear term, *P* = 0.0029 quadratic term). Associations of GA at birth with MS ((g)* P* for trend =0.0083), BP ≥ 90th percentile ((h)* P* for trend =0.0255), and TG > 110 mg/dL ((i)* P* for trend =0.0435). Panels (a), (c), and (e) show that, as the value of the perinatal variable increases, it also increases the prevalence of obesity or WC ≥ 90th percentile at 10–14 years of age. (b), (d), (g), (h), and (i) behave inversely; as the value of the perinatal variable increases, prevalence of stunting, BP ≥ 90th percentile, MS, or TG > 110 mg/dL decreases. Panel (f) shows a U-shaped behavior, denoting that the lowest and highest values of BL have the highest prevalence of BP ≥ 90th percentile, Puente Alto, Chile, 2009–2011. BW, birth weight; BL, birth length; GA, gestational age; WC, waist circumference; BP, blood pressure; MS: metabolic syndrome; TG, triglycerides; perc, percentile.

Several associations were observed between perinatal variables and the outcome variables studied (Figure 1). Positive trends were observed between BW and obesity and between BW and WC ≥ 90th percentile; BL was also positively associated with obesity. Negative associations were observed between BW and stunting and between BW and BP ≥ 90th percentile. GA was negatively associated with MS, BP ≥ 90th percentile, and TG ≥ 110 mg/dL; two other negative associations were almost significant: GA with IR (*P* for trend of 0.0893) and GA with WC ≥ 90th percentile (*P* for trend of 0.0733). In addition, a U-shaped association between BL and BP ≥ 90th percentile was observed. Another association was found between BL and stunting (*P* for trend < 0.0001); this association was not included in Figure 1.

Table 3 presents crude ORs for the associations described above; bold numbers correspond to significant OR values. Adjusted ORs for %FM and adjusted ORs for %FM, sex, and Tanner stage are also presented.

The lowest prevalence of obesity and WC ≥ 90th percentile at school-age was observed within the 2500–2999 g BW category and was therefore considered as the reference level. This prevalence increased with BW categories, almost tripling the odds for obesity and almost doubling the odds for WC ≥ 90th percentile at the highest category of BW. Adjustments for %FM increased most ORs. Adjustment for %FM, sex, and Tanner stage for obesity maintained similar values. Adjustments for %FM and for %FM, sex, and Tanner stage showed a slight increase of OR values for WC ≥ 90th percentile in the <2500 g BW category.

BW had an inverse association with the prevalence of stunting. Crude ORs in the 3500 g and over categories were protective; those values did not change after adjustments for %FM but disappeared after further adjustment for sex and Tanner stage.

BW did not show an influence on BP ≥ 90th percentile when assuming the 2500–2999 g BW category as reference (Table 3). However, when selecting the BW category with the lowest prevalence of BP ≥ 90th percentile as reference, that is, 3500–3999 g, the lowest BW category, that is, <2500 g, presented a higher risk for BP ≥ 90th percentile: OR 1.80 (95CI: 1.12–2.90); no changes were observed after adjustments.

BW had an inverse influence on IR prevalence. Crude ORs in the 3000 g and over categories were protective; those values were stable after adjustments.

52-53 cm BL was the category with the lowest prevalence of BP ≥ 90th percentile at school-age and was considered as the reference level. Both, the lowest and highest categories of BL doubled the risk of BP ≥ 90th percentile, depicting a U-shaped behavior. Adjustments for %FM and for %FM, sex, and Tanner stage maintained these ORs only for BL < 48 cm.

BL categories below 50 cm were protective for obesity. ORs declined and were more protective when adjusting for %FM. These associations disappeared when further adjusting for sex and Tanner stage.

52-53 cm BL was the category with the lowest prevalence of stunting at school-age; BL ≥ 54 cm category did not present stunted children. OR values < 50 cm showed a risk of at least 5 times the observed in the reference category. Adjustments for %FM and for %FM, sex, and Tanner stage maintained these ORs.

One BL category, that is, 48-49 cm, increased the risk for HOMA-IR just when adjusting for %FM, sex, and Tanner stage.

GA ≥ 41 weeks was the category with the lowest prevalence of BP ≥ 90th percentile, MS, and TG ≥ 110 mg/dL at school-age and was considered the baseline level. Both, crude and adjusted ORs of prematurity, defined as GA ≤ 37 weeks, behaved in a similar way showing the highest figures for BP ≥ 90th percentile, MS, and TG ≥ 110 mg/dL. No associations were found for GA and IR.

## 4. Discussion

This is the first study focusing upon associations between perinatal factors and nutritional status, MS, and IR in Chilean children. We observed that prenatal growth influences obesity characteristics, MS, IR, and stunting. In general, prenatal growth seemed to be more important in predicting these associations than present body composition because adjustments for present fat mass increased or maintained the most prenatal growth influences. Small body size at birth was associated with elevated BP, IR, and MS, while a large body size at birth was associated with measurements of obesity. The undeniable role of prenatal growth on metabolic outcomes appears, even in school-aged children, whose metabolic damages are only developing.

The finding of a U-shaped association between BL and BP ≥ 90th percentile is consistent with the well-documented associations between both low and high values of BW and BL with many perinatal health indicators in previous reports [19, 35].

The associations of BW with a higher prevalence of obesity or increased adiposity have been observed in previous studies [18, 36]. In our report there was also a nonsignificant tendency towards a J-shaped association which according to other studies may be stronger and significant at later ages. For example, the 1958 British birth cohort study showed that the association between BW and BMI was positive and weak becoming more J-shaped with increasing age; BMI was assessed at 7, 11, 16, 23, and 33 years in that study [37]. This inverse association between low BW and obesity and WC > 90th percentiles has been explained by Hales and Barker with the “thrifty phenotype” hypothesis. This proposes a greater susceptibility of the newborns with low BW to develop IR in order to survive intrauterine malnutrition and determine adaptive responses as diet preference to high-fat, hyperphagia, decreased muscle development, and large deposit of visceral adipose tissue [38].

BL was positively associated with obesity, probably reflecting the well-known previously reported finding that weight and height are positively correlated during adolescence but not during adulthood [9].

GA was inversely associated with important metabolic risk factors, BP ≥ 90th percentile, TG ≥ 110 mg/dL, WC ≥ 90th percentile, and MS as a whole, in these children. As previously mentioned prematurity was defined in this study as ≤37 weeks following recent national recommendations [35]. This fact leads to estimating a higher proportion of risky premature births at the national level; live births with 37 weeks, estimated to be in 6%, would add the proportion traditionally defined as premature births with GA < 37 w, which are now reaching 6.13% for 2011 [16, 35]. The incidence of preterm is increasing worldwide and, as previously mentioned, has been associated with increased BP and IR [11, 12]. A lower GA necessarily implies a lower fetal growth as estimated by BW and BL; much more attention should be given to its prevention.

National proportions of BL < 50 cm and ≥54 cm are now reaching 44% and 2.5%, respectively, meanwhile national proportions of BW < 3000 g and ≥4000 g are now reaching 21.8% and 9%, respectively [16, 18, 19, 35]. It can be inferred that BL < 50 cm is the most frequent factor of risk in this study. Anyway, there is a clear need to avoid extreme values of perinatal variables.

Most of these results confirmed observations of a previous study [6]. However, this is the first Chilean sample that shows U- or J-shaped nature of some of the associations [39–46]. All previous studies had a lower sample size than the present study and also less power to detect J- or U-shaped associations between BL and BP. Besides, the other seven quoted reports apparently did not search for the possible U-shaped associations of BW with obesity or WC; anyway, in most of them infants or children were too young to show that kind of associations as discussed previously [37].

There are some weaknesses in our study that need to be addressed. The sample comes from all public schools in Puente Alto, the county with the highest number of inhabitants in Chile reaching more than 600,000 [16]. Cases that did not sign the informed consent were mostly boys. However the final study sample consisted of 52% females and 48% males, with a similar sex distribution to all live births at the national level [16]. No significant differences were found between the students who signed and did not sign the informed consent with respect to their prenatal and maternal characteristics. These findings suggest that there was not a biased selection of study subjects due to follow-up losses. Another limitation of this study is the lack of consensus on the diagnostic classifications of MS in children older than 6 years [18, 47, 48]; this difficulty is due to the possible influences of growth and puberty [49].

Body composition at school-age would need to be associated with the same information at birth and also during pregnancy [50]. Moreover, other maternal data could provide light on the role of nutritional status and certain pathologies on the observed results of this study. Although there is a known association between nutrition during pregnancy, infancy, and childhood with metabolic disorders, specific studies in different settings are needed [1–4].

## 5. Conclusions

Prenatal growth is related to MS, IR, and nutritional status in this large sample of 3290 Chilean children. Fetal growth seemed to be generally more important in these associations than in present body composition because adjustments for present fat mass increased or maintained the most prenatal growth influences.

The most significant results may include association of anthropometry at birth with MS, obesity, BP ≥ 90th percentile, and stunting. Other results confirmed previous observations regarding high risk of obesity and high risk of WC ≥ 90th percentile in newborns who weighed ≥ 4000 g at delivery. BW had a strong negative association with BP ≥ 90th percentile as previously reported. Interestingly, BL < 50 cm was associated with at least ~30% reduction in the risk of obesity and at least five times increase in the OR of stunting. The lowest and highest categories of BL were associated with a 2-fold increase in the risk of high BP. On the other hand, children who were born ≤37 weeks exhibited higher risk for BP ≥ 90th percentile, hypertriglyceridemia, and MS.

## Figures and Tables

**Table 1:** Anthropometric and perinatal characteristics (mean ± SD) by sex in 3290 children from Puente Alto, Chile, 2009–2011.

Variable	Total	Girls	Boys	*P* value
	(*n* = 3290)	(*n* = 1711)	(*n* = 1579)	
Age (years)	11.4 ± 1.0	11.4 ± 1.0	11.5 ± 1.0	<0.001
Weight (kg)	43.9 ± 11.2	44.4 ± 11.1	43.4 ± 11.3	0.018
Height (cm)	146.5 ± 8.1	146.8 ± 7.7	146.2 ± 8.5	0.042
BMI (kg/m^2^)	20.3 ± 3.9	20.4 ± 3.9	20.1 ± 3.9	0.038
*z*-BMI	0.59 ± 1.1	0.59 ± 1.0	0.58 ± 1.1	ns
%FM	25.0 ± 11.4	27.0 ± 12.2	22.8 ± 10.1	<0.001
BW (g)	3350 ± 524	3292 ± 512	3412 ± 529	<0.001
BL (cm)	49.6 ± 2.4	49.2 ± 2.3	49.98 ± 2.4	<0.001
GA (w)	38.9 ± 1.8	38.9 ± 1.9	38.8 ± 1.7	ns

BMI, body mass index; %FM, percentage of fat mass; BW, birth weight; BL, birth length; GA, gestational age; ns, nonsignificant; SD, standard deviation.

**Table 2:** Anthropometric and perinatal characteristics [*n* (%)] by sex in 3290 children from Puente Alto, Chile, 2009–2011.

Variable	Total	Girls	Boys	*P* value
	(*n* = 3290)	(*n* = 1711)	(*n* = 1579)	
Obesity (BMI ≥ 95 percentile)	527 (16.0)	227 (13.3)	300 (19.0)	0.002
Stunting (stature-for-age < 5th percentile)	120 (3.6)	63 (3.7)	57 (3.1)	0.049
MS (≥3 components)	239 (7.3)	150 (8.8)	89 (5.6)	0.001
HDL-C ≤ 40 mg/dL	555 (16.9)	324 (18.9)	231 (14.6)	0.001
TG ≥ 110 mg/dL	878 (26.7)	530 (31.0)	348 (22.0)	<0.001
SBP (mm Hg) ≥ 90th percentile	349 (10.6)	199 (11.6)	150 (9.5)	ns
DBP (mm Hg) ≥ 90th percentile	24 (0.73)	17 (1.0)	7 (0.4)	ns
BP (mm Hg) ≥ 90th percentile	354 (10.8)	201 (11.7)	153 (9.7)	ns
WC (cm) ≥ 90th percentile	697 (21.2)	405 (23.7)	292 (18.5)	<0.001
Glucose (mg/dL) ≥ 100 mg/dL	235 (7.1)	96 (5.6)	139 (8.8)	<0.001
IR (HOMA ≥ 90th percentile)	838 (25.5)	424 (25.2)	414 (26.4)	ns

BMI, body mass index; MS, metabolic syndrome; HDL-C, high-density lipoprotein; TG, triglycerides; SBP, systolic blood pressure; DBP, diastolic blood pressure; BP, blood pressure; WC, waist circumference; HOMA, homeostasis model assessment; IR, insulin resistance; ns, nonsignificant.

**Table 3:** Trends of the main outcomes in school-age children with perinatal variables. Prevalence and crude ORs and ORs adjusted by %FM, Puente Alto, Chile, 2009–2011.

BW (g)	*n*	Obesity (%)	OR	95% CI	OR∗	95% CI	OR∗∗	95% CI

<2500	161	11.8	1.20	0.69–2.11	1.81	0.81–4.05	2.11	0.89–4.98
2500–2999	500	10.0	1.00	Baseline	1.00	Baseline	1.00	Baseline
3000–3499	1335	14.0	**1.47**	**1.05–2.04**	**2.00**	**1.28–3.13**	**2.06**	**1.27–3.35**
3500–3999	992	19.8	**2.22**	**1.59–3.09**	**3.55**	**2.26–5.58**	**3.07**	**1.87–5.04**
≥4000	302	24.8	**2.97**	**2.01–4.40**	**3.94**	**2.26–6.87**	**2.81**	**1.52–5.20**

BW (g)	*n *	WC ≥ 90th percentile (%)	OR	95% CI	OR∗	95% CI	OR∗∗	95% CI

<2500	161	19.9	1.18	0.75–1.85	**2.64**	**1.29–5.39**	**2.65**	**1.29–5.45**
2500–2999	500	17.4	1.00	Baseline	1.00	Baseline	1.00	Baseline
3000–3499	1335	19.7	1.17	0.89–1.52	**1.53**	**1.03–2.29**	1.49	0.99–2.23
3500–3999	992	23.5	**1.46**	**1.11–1.92**	**1.94**	**1.29–2.93**	**1.79**	**1.18–2.72**
≥4000	302	27.2	**1.77**	**1.26–2.49**	**1.75**	**1.03–2.98**	1.54	0.89–2.65

BW (g)	*n *	Stunting (%)	OR	95% CI	OR∗	95% CI	OR∗∗	95% CI

<2500	161	6.8	1.11	0.54–2.26	1.02	0.5–2.08	0.65	0.37–1.13
2500–2999	500	6.2	1.00	Baseline	1.00	Baseline	1.00	Baseline
3000–3499	1335	4.3	0.68	0.43–1.06	0.67	0.42–1.05	0.75	0.47–1.18
3500–3999	992	1.7	**0.26**	**0.15–0.48**	**0.27**	**0.15–0.50**	1.08	0.71–1.63
≥4000	302	1.3	**0.20**	**0.07–0.58**	**0.22**	**0.08–0.63**	**1.29**	**0.54–3.12**

BW (g)	*n *	BP ≥ 90th percentile (%)	OR	95% CI	OR∗	95% CI	OR∗∗	95% CI

<2500	161	15.5	1.35	0.81–2.23	1.41	0.85–2.34	1.42	0.85–2.35
2500–2999	500	12.0	1.00	Baseline	1.00	Baseline	1.00	Baseline
3000–3499	1335	10.9	0.89	0.65–1.23	0.90	0.65–1.24	0.90	0.65–1.25
3500–3999	992	9.3	0.75	0.53–1.06	0.73	0.52–1.03	0.74	0.52–1.05
≥4000	302	10.6	0.87	0.55–1.37	0.80	0.51–1.27	0.82	0.52–1.30

BW (g)	*n *	IR	OR	95% CI	OR∗	95% CI	OR∗∗	95% CI

<2500	161	24.8	0.75	0.5–1.12	0.83	0.53–1.30	0.82	0.52–1.29
2500–2999	500	30.6	1.00	Baseline	1.00	Baseline	1.00	Baseline
3000–3499	1335	24.5	**0.74**	**0.59–0.92**	**0.70**	**0.55–0.90**	**0.66**	**0.51–0.85**
3500–3999	992	25.3	**0.77**	**0.61–0.98**	**0.67**	**0.51–0.87**	**0.60**	**0.46–0.78**
≥4000	302	28.2	0.89	0.65–1.22	**0.67**	**0.47–0.95**	**0.57**	**0.40–0.82**

BL (cm)	*n *	BP ≥ 90th percentile (%)	OR	95% CI	OR∗	95% CI	OR∗∗	95% CI

<48	457	16.6	**2.22**	**1.49–3.31**	**2.25**	**1.51–3.35**	**2.22**	**1.48–3.33**
48-49	976	10.9	1.36	0.94–1.97	1.34	0.93–1.95	1.33	0.91–1.94
50-51	1264	9.3	1.15	0.80–1.65	1.12	0.78–1.62	1.12	0.77–1.61
52-53	522	8.2	1.00	Baseline	1.00	Baseline	1.00	Baseline
≥54	71	15.5	**2.04**	**1.00–4.17**	1.93	0.94–3.96	1.93	0.94–3.96

BL (cm)	*n *	Obesity (%)	OR	95% CI	OR∗	95% CI	OR∗∗	95% CI

<48	457	12.3	**0.64**	**0.45–0.92**	**0.38**	**0.23–0.62**	0.65	0.37–1.13
48-49	976	13.3	**0.71**	**0.53–0.95**	**0.43**	**0.29–0.66**	0.75	0.47–1.18
50-51	1264	18.2	1.03	0.79–1.34	0.81	0.56–1.18	1.08	0.71–1.63
52-53	522	17.8	1.00	Baseline	1.00	Baseline	1.00	Baseline
≥54	71	25.4	1.57	0.88–2.80	1.29	0.56–2.96	1.29	0.54–3.12

BL (cm)	*n *	Stunting (%)	OR	95% CI	OR∗	95% CI	OR∗∗	95% CI

<48	457	9.2	**8.70**	**3.66–20.67**	**8.64**	**3.63–20.57**	**8.74**	**3.65–20.94**
48-49	976	5.2	**4.74**	**2.02–11.12**	**4.86**	**2.07–11.42**	**4.98**	**2.11–11.75**
50-51	1264	1.2	1.45	0.58–3.62	1.51	0.6–3.76	1.52	0.61–3.79
52-53	522	1.2	1.00	Reference	1.00	Reference	1.00	Reference
≥54	71	0.0	—		—		—	

BL (cm)	*n *	IR	OR	95% CI	OR∗	95% CI	OR∗∗	95% CI

<48	457	25.8	1.12	0.84–1.49	1.15	0.83–1.58	1.38	0.99–1.91
48-49	976	27.4	1.21	0.95–1.55	1.19	0.91–1.56	**1.40**	**1.06–1.84**
50-51	1264	25.9	1.12	0.88–1.42	1.05	0.81–1.36	1.13	0.87–1.48
52-53	522	23.8	1.00	Baseline	1.00	Baseline	1.00	Baseline
≥54	71	28.2	1.26	0.72–2.19	1.04	0.56–1.94	1.00	0.53–1.85

GA (w)	*n *	BP ≥ 90th percentile	OR	95% CI	OR∗	95% CI	OR∗∗	95% CI

≤37	431	13.7	**1.73**	**1.09–2.75**	**1.74**	**1.09–2.78**	**1.77**	**1.11–2.82**
38	685	11.5	1.42	0.91–2.21	1.42	0.91–2.21	1.43	0.92–2.22
39	928	10.9	1.33	0.87–2.04	1.36	0.88–2.08	1.37	0.89–2.10
40	889	9.6	1.15	0.75–1.78	1.17	0.76–1.82	1.18	0.76–1.83
≥41	357	8.4	1.00	Baseline	1.00	Baseline	1.00	Baseline

GA (w)	*n *	MS (%)	OR	95% CI	OR∗	95% CI	OR∗∗	95% CI

≤37	431	10.2	**2.14**	**1.21–3.78**	**2.37**	**1.28–4.39**	**2.46**	**1.33–4.55**
38	685	8.2	1.68	0.97–2.90	1.76	0.97–3.17	1.78	0.99–3.22
39	928	6.5	1.30	0.76–2.24	1.51	0.84–2.70	1.56	0.87–2.79
40	889	6.9	1.39	0.81–2.38	1.55	0.86–2.77	1.58	0.88–2.84
≥41	357	5.0	1.00	Baseline	1.00	Baseline	1.00	Baseline

GA (w)	*n *	TG ≥ 110 mg/dL (%)	OR	95% CI	OR∗	95% CI	OR∗∗	95% CI

≤37	431	30.6	**1.41**	**1.03–1.94**	**1.48**	**1.06–2.07**	**1.52**	**1.09–2.13**
38	685	27.0	1.18	0.88–1.59	1.19	0.87–1.63	1.21	0.88–1.65
39	928	25.2	1.08	0.81–1.44	1.14	0.85–1.54	1.16	0.86–1.57
40	889	27.2	1.20	0.90–1.59	1.28	0.95–1.73	1.30	0.96–1.75
≥41	357	23.8	1.00	Baseline	1.00	Baseline	1.00	Baseline

*OR adjusted by %FM.

∗∗OR adjusted by %FM, sex, and Tanner stage.

WC, waist circumference; BP, blood pressure; MS, metabolic syndrome; TG, triglycerides; BP, blood pressure; HOMA-IR: homeostasis model assessment index-insulin resistance; BW, birth weight; BL, birth length; GA, gestational age; OR, odds ratio; CI, confidence interval, %FM: percentage of fat mass.
